# Supplementary material for: Influence of Annealing on Mechanical Behavior of Alumina-Tantala Nanolaminates
Source: Materials (Basel). 2023 Apr 19;16(8):3207. doi: 10.3390/ma16083207 (PMC10143553; doi:10.3390/ma16083207)
Supplement: Supplementary file 1 [file materials-16-03207-s001.zip › materials-2340791-supplementary.pdf]

materials

Article

# Influence of Annealing on Mechanical Behavior of Alumina-Tantala Nanolaminates

**Helle-Mai Piirsoo*, Taivo Jõgiaas, Kaupo Kukli and Aile Tamm**

Institute of Physics, University of Tartu, W. Ostwaldi str 1, 50411 Tartu, Estonia
* Correspondence: helle-mai.piirsoo@ut.ee (H.-M.P.)

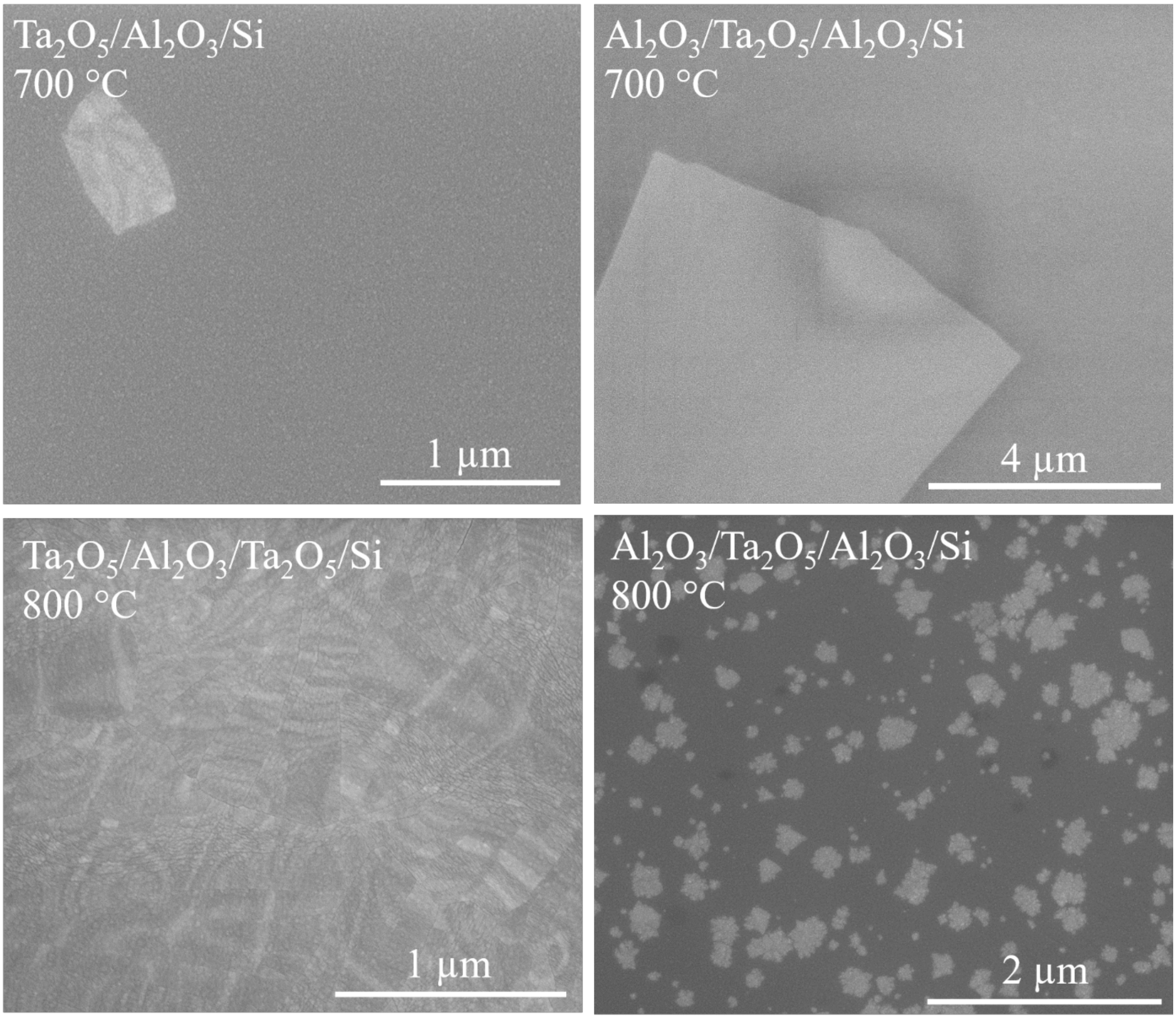


**Figure S1.** SEM images of surfaces of $Ta_2O_5/Al_2O_3/Si$ and $Al_2O_3/Ta_2O_5/Al_2O_3/Si$ after annealing at 700 °C and $Ta_2O_5/Al_2O_3/Ta_2O_5/Si$ and $Al_2O_3/Ta_2O_5/Al_2O_3/Si$ after annealing at 800 °C.
